# From Translation to Protein Degradation as Mechanisms for Regulating Biological Functions: A Review on the SLRP Family in Skeletal Tissues

**DOI:** 10.3390/biom10010080

**Published:** 2020-01-03

**Authors:** Jérémie Zappia, Marc Joiret, Christelle Sanchez, Cécile Lambert, Liesbet Geris, Marc Muller, Yves Henrotin

**Affiliations:** 1 Bone and Cartilage Research Unit, Arthropôle Liège, Center for Interdisciplinary research on Medicines (CIRM) Liège, Liège University, Institute of Pathology, CHU Sart-Tilman, 4000 Liège, Belgium; jzappia@uliege.be (J.Z.); christelle.sanchez@uliege.be (C.S.); cecile.lambert@uliege.be (C.L.); 2 Biomechanics Research Unit, B34 GIGA-R, In Silico Medicine, Liège University, CHU Sart-Tilman, 4000 Liège, Belgium; marc.joiret@uliege.be (M.J.); Liesbet.Geris@uliege.be (L.G.); 3 Laboratory for Organogenesis and Regeneration (LOR), GIGA-Research, Liège University, Avenue de l’Hôpital, B-4000 Liège, Belgium; m.muller@uliege.be; 4 Physical therapy and Rehabilitation department, Princess Paola Hospital, Vivalia, B-6900 Marche-en-Famenne, Belgium; 5 Artialis SA, GIGA Tower, Level 3, CHU Sart-Tilman, 4000 Liège, Belgium

**Keywords:** small leucine-rich proteoglycans, codon usage, post-translational event, glycosaminoglycan, glycosylation, catabolism

## Abstract

The extracellular matrix can trigger cellular responses through its composition and structure. Major extracellular matrix components are the proteoglycans, which are composed of a core protein associated with glycosaminoglycans, among which the small leucine-rich proteoglycans (SLRPs) are the largest family. This review highlights how the codon usage pattern can be used to modulate cellular response and discusses the biological impact of post-translational events on SLRPs, including the substitution of glycosaminoglycan moieties, glycosylation, and degradation. These modifications are listed, and their impacts on the biological activities and structural properties of SLRPs are described. We narrowed the topic to skeletal tissues undergoing dynamic remodeling.

## 1. Introduction

The extracellular matrix (ECM) is a three-dimensional network of extracellular macromolecules that provides structural and biochemical support to surrounding cells. More than a physical support tissue, the ECM is a dynamic structure that not only depends on but also regulates cell behavior and functions. Any change in its composition may reflect a disease state or may initiate a disease [1,2,3]. Glycoproteins, collagens, and proteoglycans are the main components of the ECM, and they constitute the “core matrisome” encompassing almost 300 proteins in mammals. This matrisome contains 35 proteoglycans including small leucine-rich proteoglycans (SLRPs). The majority of SLRPs control ECM assembly by regulating collagen fibrillogenesis [4,5,6]. In addition, SLRPs are able to control and interact with various cytokines in the extracellular space and bind to cellular receptors [7,8,9,10].

In this review, the SLRP family’s codon usage was investigated. The codon usage is commonly associated to pathological or stress conditions. In many coding sequences, the usage patterns of rare versus common synonymous codons are nonrandom and under selection pressure. Synonymous rare codons can enhance co-translational protein folding, increasing the likelihood of forming the native protein structure and suppressing alternatively folded structures [11,12,13,14]. They increase the time available for post-translational chemical reactions to occur on specific residues upstream in the oriented sequence (N-terminal toward C-terminal), even before the protein translation is fully completed, adding an extra layer to the dynamic translational control [15]. Here, the coding DNA sequence (CDS) of 17 SLRP proteins, for which the human CDS is known, were data-mined for their codon usage for seven amino acid residues deemed relevant to characterize the SLRP family.

Post-translational modifications are defined as the set of modifications that happen on the core protein such as the addition of a chemical group or the cleavage of a sequence of amino acid residues. The regulation of post-translational modifications was shown to play a role in the modulation of cell biological functions [16]. There are more than 200 different types of post-translational modifications, the most common within SLRPs being glycanation, glycosylation, and enzymatic or oxidative degradation. Changes in SLRPs are associated with certain pathologies, and the pattern of these modifications could be used as a biomarker to diagnose diseases and predict their progression [6,9].

This review describes the impact of the SLRP codon usage pattern and their post-translational modifications on the homeostasis of skeletal tissues. These two regulatory pathways are key cellular mechanisms for adaptation to stress and pathological conditions. Herein, the human skeletal system is defined as all the bony, ligamentous, fibrous and cartilaginous elements that together make up the skeleton and its attachment.

## 2. The SLRP Family: Classification and Structure

**Figure 1:**
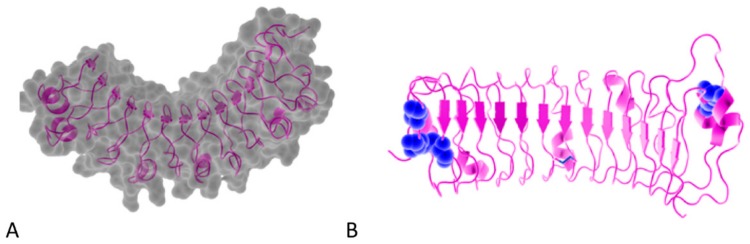
(**A**). Osteomodulin in 3D view (PDB id: 5YQ5) with PyMol showing the horseshoe shape with 13 β-sheets on the concave side and 13 α-helices on the convex side. (**B**). Osteomodulin in 3D view (PDB id: 5YQ5) with PyMol showing cystein residues (in blue) of the protein near the N-terminal (C66, C68–C78 on the left) and C-terminal end (C321–C353 on the right downstream of the ear-repeat). The secondary structure’s graphical representations showing the small leucine-rich proteoglycans (SLRPs) salient features were carried out with the PyMol software (PyMOL^TM^ 2.3.1—Incentive Product Copyright Schrodinger, LLC) and using home-made Python scripts to display relevant amino acids in chosen colors.

Proteoglycans are complex macromolecules composed of a core protein covalently decorated by glycosaminoglycan (GAG) chains on serines or threonines. The small core protein has a molecular weight between 36 kDa and 77 kDa and is characterized by a variable number of central leucine-rich repeat (LRR) domains, each made of one α-helix and one β-strand. SLRPs are solenoid-like proteins with a horseshoe shape. Their concave face is formed of β-sheets, while the convex surface is formed of α-helices, where each LRR provides one β-sheet and one α-helix that correspond to one turn of the solenoid-like structure, the hydrophobic residues of the LRR facing inside. This structure is favorable to protein–protein interaction where the inner concave face is ideal to capture a ligand [17,18,19] (Figure 1A). The consensus sequence of LRR is LXXLXLXXNXL, where L is a leucine, an isoleucine, a valine, or another hydrophobic amino acid; N is an asparagine; and X is any amino acid. LRRs are composed of variable amino acid sequences across the SLRP family, ranging in size from 20 to 29 residues, with 24 residues being the most representative [18,19]. The N and C-terminal regions of the protein flanking the central LRR domain are rich in cysteine residues. The N-terminal domain contains four cysteines spaced by a varying number of amino acids. This N-terminal domain shows variability within the SLRP family. The cysteines present in this cluster form a disulfide bond between the first LRR and a β-hairpin. The C-terminal domain generally contains two cysteines [20]. The C-terminal end includes the two C-terminal LRRs with the penultimate one called the ear-repeat. The ear-repeat is the longest LRR that is laterally extended from the main axis of the core protein to form the ear [18,19] (Figure 1B). Ear-repeats are thought to be involved in ligand recognition and in the structural folding of the protein. As an example, a truncated decorin lacking C-terminal end residues is associated with congenital stromal corneal dystrophy. This mutated decorin is insoluble and retained in the cell, leading to an endoplasmic reticulum stress and an unfolded protein response [21].

The SLRP family is composed of 18 members that have been classified into five distinct classes according to their structural and functional properties and their chromosomal organization. The five classes are divided into canonical and non-canonical classes. The presence of the ear-repeat is a feature of all the canonical SLRPs. The classes I–III (13 members) include the canonical SLRPs and the classes IV–V (5 members) include the non-canonical SLRPs [9,19] (Table 1).

Glycosaminoglycans (GAGs) are long linear polysaccharides made of repeated dissacharide units consisting of an amino sugar (N-acetylgalactosamine or N-acetylglucosamine) and an uronic acid (glucuronic acid or iduronic acid), on the basis of which they can be classified. They are synthetized through a complex enzymatic pathway starting from the addition of four monosaccharides (xylose, galactose, galactose, and glucuronic acid) on the hydroxyl group of a serine, except for the keratan sulfate (see below). This tetrasaccharide is the linker to which the different additional units are attached to form the diverse GAGs; additional diversity is generated through epimerization, sulfation, and deacetylation [22]. Keratan sulfate addition results either from an N or an O-glycosylation via three different possible linkers, from which the chain elongation occurs. Interestingly, keratan sulfate might be associated with a tyrosine sulfation pattern, as the two coincide in the same SLRPs each time. GAG biosynthesis and modifications mainly take place in the Golgi apparatus [22,23]. GAGs are subject to degradation within lysosomes where different enzymes such as exohydrolase and endoenzymes catabolize them to oligosaccharides with further desulfation steps [24,25].

Many GAG side chains may be attached to SLRP core proteins, mainly to the N-terminal part. These GAG modifications are often similar for an SLRP class and therefore can be used for their characterization (Table 1). GAG chains are involved in the structure, the conformational stability, Ref. [26] and the secretory process of SLRPs [27].

## 3. SLRP Codon Usage Patterns May Fine-Tune Selective Translation Pathways during Cellular Stress Conditions

The SLRP family members share common structural features as described in the classification and structure section and partially illustrated in Table 1. Here, we conducted a biostatistical and bioinformatics exploratory analysis of the SLRP family members based on the codon usage of the genes encoding 17 members of the family.

Biases in codon usage have been documented to play a role in the translational fine-tuning of protein expression levels for cells undergoing stress conditions. This fine-tuning involves enzymatic modifications (i.e., reprogramming) of specific t-RNAs on their U34 wobble-base [59]. These t-RNA U34 wobble-base modifications affect the elongation rate during translation for transcripts that have a specific pattern of codon usage, resulting in a selective increase in the translation of specific proteins by the cytoplasmic pool of ribosomes. A well-known example [60] is the modified U34 wobble-base called mcm^5^-s^2^-U34 (methoxy-carbonyl-methyl-5, thio-2 uridine 34), which is catalyzed by three successive enzymes targeting the original uridine t-ribonucleobase (U34-t-RNA): acetyltransferase elongator (Elp1-6), methyltransferase TRM9-like domain of alkylation repair homolog (ALKBH8), and the cytosolic thiouridylase homolog 1 and 2 (Ctu1/2). This modified t-RNA mcm^5^-s^2^-U34 wobble-base will eventually base pair to the transcript cognate codons whose third base ends preferentially with an A instead of a G, thus resulting in a privileged base pairing with the rarely used codons for lysine, glutamine, and glutamate.

In yeasts, this mechanism has been shown to enhance the translation of proteins involved in hypoxic stress responses [61]. In melanoma cancer cells, it has been shown to enhance the expression of HIF1-α and of other proteins enriched in the codon usage patterns that happen to be promoted in tumor cells resistant to targeted therapy [62].

Even in the absence of enzymatic tRNA modifications, the usage of rare codons may be required to slow down the translating ribosome to increase the available time for proper folding of the nascent protein or to increase the likelihood of chemical additions (e.g., hypothetical glycanation tagging) on specific residues located upstream in the sequence being translated for later post-translational purposes.

The coding DNA sequences (CDS) of 17 SLRP proteins (n = 17 observations) were data-mined for their codon usage of seven amino acid residues deemed relevant to characterize the SLRP family: cysteine (C:Cys), lysine (K:Lys), glutamine (Q:Gln), glutamate (E:Glu), asparagine (N:Asn), aspartate (D:Asp), and of course leucine (L:Leu). The genetic code degeneracy associated to these amino acid residues reads as follows: cysteine, two codons: UGC, UGU; lysine, two codons: AAA, AAG; glutamine, two codons: CAA, CAG; glutamate, two codons: GAA, GAG; asparagine, two codons: AAU, AAC; aspartate, two codons: GAU, GAC; and finally leucine, six codons: UUA, UUG, CUA, CUC, CUG, and CUU. The subset of all these investigated codons contains 18 codons (p = 18 variables).

**Figure 2:**
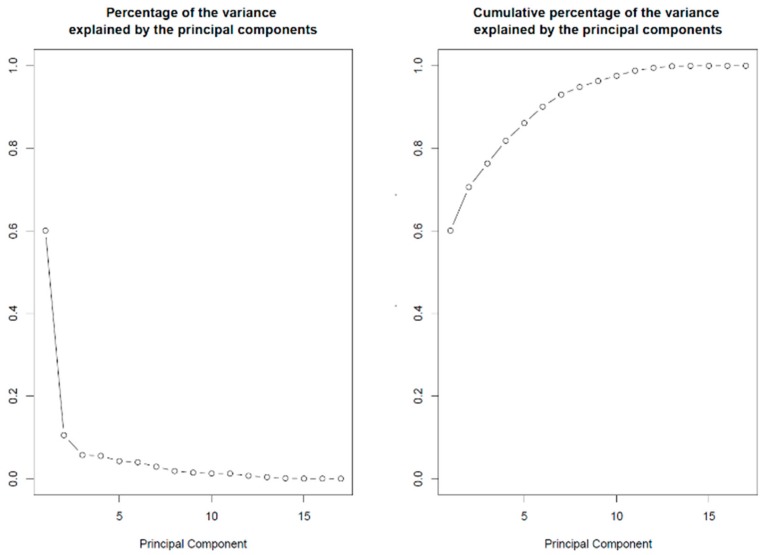
Percentage of variance explained by the PCs. Left panel: The first two components explain 60.08% and 10.53% of the variance, respectively. Right panel: Cumulative percentage of the variance explained. The first two components explain 60.08 + 10.53 = 70.6% of the variance in codon usage between SLRP family members’ proteins.

The coding DNA sequences (CDS) of 17 members of the SLRP family (all SLRP members except the class I protein ECMX, whose CDS sequence was not found) were extracted from the Ensembl human genome repository (GRCh38.p12 assembly) in FASTA format. The complete coding sequences were collected with the longest possible open reading frame (ORF) starting from start codon AUG and ending at the most downstream occurrence of one of the three possible stop codons (UAA, UAG, UGA). We processed each sequence to count the total number of codons in the longest open reading frame, to count the codon usage of the seven aforementioned amino acid residues and to compute the codon usage frequencies of the corresponding 18 codons under investigation with a home-made Python program using BioPython modules (importing SeqIo, IUPAC, codonTable and suffix trees). The data were exported and then retrieved in R for further statistical analysis. The codon frequencies revealed different bias patterns in codon usage between the SLRP family members, on which an unsupervised statistical learning analysis was performed [63]. The results of the principal component analysis (PCA) are presented below. The PCA was conducted with the prcomp function in R and graphically represented with the biplot function in R (both functions in software package ‘stats’ in R version 3.5.2. R Core Team, 2014. https://www.r-project.org/). Figure 2 displays the percentage of the variance that is explained by the principal components (PCs). The first two PCs explain 60.08% and 10.53% of the variance, while together they explain more than 70.6% of the data variance.

In a graphical representation, orthogonal loading vectors tend to be less correlated with each other than co-linear or almost co-linear loading vectors (vectors pointing in the same, or opposite direction), which are interpreted as highly correlated variable features.

**Figure 3:**
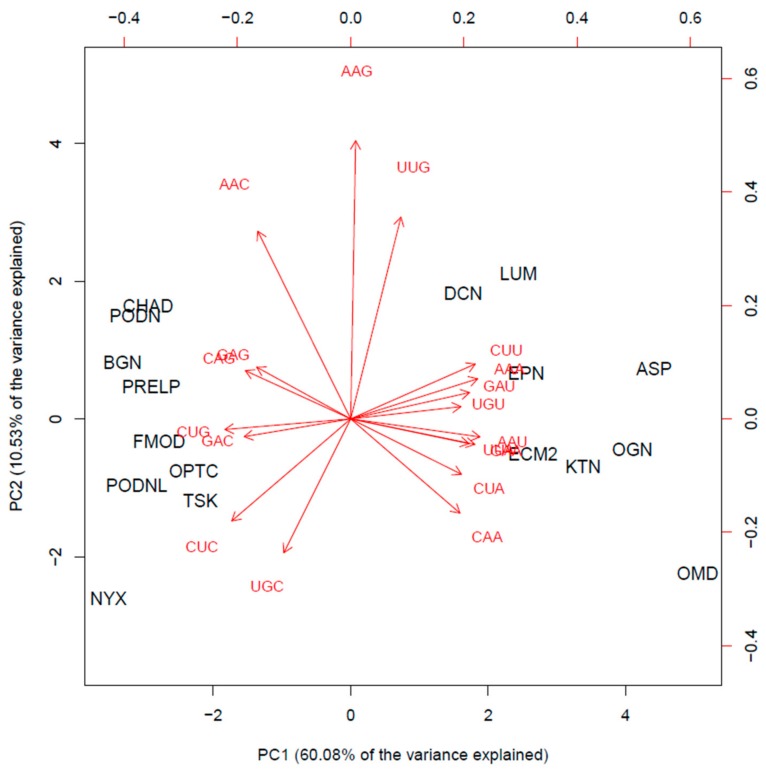
Biplot: scores of the 17 members of the SLRP family (in black) projected on the first two PCs (bottom and left first two PC scores), and red arrows indicate the loading vectors in the space of the codon usage frequency features (top and right axes first two PC loadings).

The biplot on Figure 3 displays a 2D representation of the scores for the 17 protein members from the SLRP family (in black) projected along the first two principal components and a representation of the 18 loading vectors for the codon usage features (red arrowed vectors). It shows that the SLRP family mainly clusters in two groups and that osteomodulin and nyctalopin appear as outlying members of the SLRP family. There seems to be at least two clusters with different codon usage patterns. In cluster 1 (left part of the biplot), the chondroadherin (CHAD), biglycan (BGN), Prelp (PRELP), fibromodulin (FMOD), opticin (OPTC), tsukushi (TSK), podocan (PODN), podocan like protein 1 (PDNL), and nyctalopin (NYX) mature mRNA transcripts all present a similarly biased codon usage pattern represented in Table 2. Cluster 2 (right part of the biplot) is made of decorin (DCN), lumican (LUM), epiphycan (EPN), asporin (ASP), ECM2, osteoglycin (OGN), keratocan (KTN) and osteomodulin (OMD). Its codon usage pattern contrasts with that of cluster 1 and is represented in Table 2.

In Cluster 2, it is noted that osteomodulin appears to have a strong codon usage bias. Indeed, leucine (L), in addition to CUU, is significantly encoded by the rare CUA, and the three K, Q, and E amino acid residues are mostly encoded by the unusual codons ending with the AA dinucleotide. Thus, their codon usage is strongly biased toward the A-ending codons, which are known to preferentially base pair with the modified mcm^5^s^2^–U34 tRNA. Therefore, we suspect that the osteomodulin protein translation rate would be enhanced in cells undergoing hypoxic stress or mechanical stress via enzymatic reprogramming of the wobble U34 in the cognate t-RNA decoding leucine, glutamine, glutamate, and lysine residues. Supplementary Figures S1 and S2 show the codon usage biased patterns contrasting osteomodulin with the other members of the SLRP family for leucine and for the triplet glutamine, glutamate, and lysine amino acids residues.

Due to a similar though less extreme codon usage bias, it is hypothesized that asporin, osteoglycin, keratocan, and ECM2 could also have their translation rate enhanced in cellular stress conditions. The occurrence of re-programming of the wobble-base U34 tRNA under stress conditions has not yet been checked experimentally in the context of skeletal or cartilaginous tissues. Experimental validation is required to investigate whether this cascade of events results in differential levels of protein expression in the SLRP family under cellular stress conditions.

## 4. Roles of SRLP GAG Moieties in Fibrillogenesis

A major feature of the SLRPs is their interaction with collagen fibrils in the ECM. Although they bind through their core protein, their GAGs are also functional actors. GAG moieties were shown to interact directly with collagen and to modulate their fibrillogenesis [64,65]. High-resolution scanning electron microscopy indicates that these interactions are periodic and GAG chains bound to the collagen surface can form interfibrillar elastic bridges and belts around fibrils. GAGs regulate the fibril diameter and the interfibrillar spacing, and they also assemble the collagen fibrils, thus generating a network that organizes the ECM [64,66,67,68]. In the interfibrillar space, a majority of GAGs is oriented spanning adjacent fibrils, while also interacting together to form antiparallel structures called “shape modules” and keeping collagen fibrils at defined distances [66,69,70,71].

Data from in vivo studies also suggest collagen fibril regulation through the presence of GAGs. The glycanation of decorin plays a critical role in the early stages of fibrillogenesis and reduces the collagen fibril diameter. In Ehlers–Danlos syndrome, the decrease of galactosyltransferase activity impairs the glycanation of decorin and biglycan, and collagen fibrillogenesis is decreased [72,73,74]. In contrast, decorin dermatan sulfate-deficient knock-in mice, where the site of GAG attachment was mutated, fail to express any variation of the collagen fibril diameter. Although we cannot exclude compensation by other SLRPs associated with dermatan sulfate in the mouse model or the lack of functional conservation of GAGs between human and mouse [75], this discrepancy indicates that more studies are required to clarify the role of SRLPs’ GAG chains.

A model has been proposed in which GAG bridges could manage mechanical stress, namely occurring in tendons that are biological structures transmitting large forces between muscles and bones. GAG bridges are supposed to play a role in force control through fibril-to-fibril interactions during loading. In addition, decorin enhances mechanical properties by reducing fibrils aggregation during polymerization [76]. Therefore, SLRPs could protect collagen fibrils against excessive mechanical strains [77,78,79,80]. However, this model is subject to controversy. Indeed, several studies have found that the enzymatic removal of GAGs did not affect the mechanical properties of collagen fibrils [71,81,82,83] except for pathological post-injury tendons [84]. Controversially, another investigation of the collagen mechanical response in both native and GAG-depleted tendons in mouse proposed a model where GAGs promote the fibrils sliding under straining conditions by isolating individual fibrils to bear the load [85]. This conflicting evidence leaves the question open about the complex functions of the GAG and SLRP extrafibrillar network in ECM assembly.

Besides direct interaction and fibril organization, GAGs have a protective role. When SLRPs are bound to collagen fibrils, their GAGs protect collagen fibrils in acidic conditions against cathepsin K, conferring on them putative functions during bone resorption [86].

## 5. SLRP GAG Moieties: Fingerprints of the Tissue Status and Active Players

The diversity of SLRP’s GAG moieties is a characteristic of the tissue and can serve to track its status. Furthermore, the same SLRP displays different forms via the absence or presence and diversity of its GAGs.

In articular cartilage, intervertebral disc, and dental tissue, biglycan and decorin may be represented under a proteoglycan or a non-proteoglycan form [87,88,89]. Immunoblots before and after the removal of glycanation have revealed two forms of biglycan lacking GAG whose abundance is low in juveniles in the articular cartilage and in the intervertebral disc but increases in adults until they become predominant. The abundance of the biglycan proteoglycan form appears to be stable through life [87,88]. However, for decorin, its non-proteoglycan form is always a minor component, even though there is also an increase related to age [88]. In cortical bone, the level of decorin glycanation decreases with age [90]. Finally, the decorin GAG length is reduced in the tendon fascicle of old mice [91].

The glycanation of fibromodulin is modified by aging, similar to decorin and biglycan. The proteoglycan form is present in juvenile human articular cartilage, and the GAG chains are shortened through the aging process until they are mostly represented by the non-proteoglycan form in mature cartilage. However, fibromodulin continues to be substituted with N-linked oligosaccharide [92].

It appears that lumican, a keratan sulfated SLRP, goes through the same age-related mechanism in human articular cartilage. The increased representation of the non-glycanated form of lumican is due to a lack of keratan sulfate synthesis by mature chondrocytes. It is interesting to point out that aggrecan conserves its keratan sulfate chains in mature cartilage, demonstrating that chondrocytes are still able to synthesize [93,94]. In human cultured chondrocytes, it was shown that growth factors/cytokines were able to modulate the GAG chain length and sulfation of lumican by regulating the cell metabolism. IL-1β induces the production of a non-glycanated lumican, whereas the length of GAG chains in glycanated forms is modulated by basic fibroblast growth factor (bFGF), insulin growth factor (IGF)-1, and transforming growth factor (TGF)β [95].

Osteomodulin presents a different pattern of changing glycosylation profiles among keratan sulfated SLRPs, in that it varies through endochondral bone formation and its biomineralization process. In non-mineralized ECM, osteomodulin is non-glycanated and N-glycosylated, whereas in mineralized ECM, the keratan sulfate modification of osteomodulin increases with bone maturation [96].

It was suggested that the changes in growth factor/cytokine synthesis with aging could be one mechanism explaining the post-translational GAG modifications of SLRPs [95]. Non-glycanated SLRPs can also result from the degradation of a glycanated precursor, as demonstrated by the N-terminal sequence analysis of SLRPs coming from intervertebral disc tissue [87].

In addition to the presence or absence of glycanation, the nature of the GAG chains substituted to the SLRP core is also changing during bone development, and therefore could be associated with different functions. In cultured alveolar bone cells, biglycan is conjugated mainly with dermatan sulfate during the cell proliferation phase and switches to only chondroitin sulfate chains during the mineralization phase. Similarly, decorin carries many dermatan sulfate chains during early bone matrix formation but later associates only with chondroitin sulfate during mineralization [97]. The same changing profiles occur in dental tissue, where in the predentine, biglycan and decorin are mostly associated with dermatan sulfate, whereas in the predentine/dentine interface, the chondroitin sulfate becomes predominant, and in the dentine itself, it is the only GAG chain identifiable [89], while keratan sulfate distribution in the predentine forms a gradient with a maximum toward the mineralization front [98]. Moreover, the position of the sulfate within the GAG also varies within these tissues, and the GAG length is longer in the dentine than in the predentine and in the dentine/predentine interface [89]. Interestingly, GAG components are active players in the mechanical properties of the dental tissues such as stiffness and ductility due to their physicochemical properties, their location, and also by modulating the collagen structure through interfibrillar bridges. GAGs are key factors in the dentin’s ability to recover under strain and deformation events in a time-dependent manner and contribute to the dental tissues’ longevity and conservation of their mechanical integrity. GAGs are also thought to play a role in the mineralization; in dentine, chondroitin sulfate may allow the mineralization process through sequestering calcium ions. These mechanisms might be displayed in other mineralized tissues [99,100,101,102,103].

The nature of the GAG can also help to discriminate a tissue, e.g., decorin and biglycan are mostly associated with dermatan sulfate in the annulus fibrosus, but with chondroitin sulfate in the cartilage end-plate [87]. Moreover, the modifications occurring on SLRPs could be used to assess the differentiation stage of cells. In C2C12 mouse cells treated with the bone morphogenetic protein (BMP)-2, modifications to the decorin GAG chains’ length take place during the differentiation from myoblast to osteoblast. In addition to being upregulated, decorin has longer GAG chains in induced cells [104].

Defective glycanation is correlated with pathological states, for instance in gerodermia osteodysplastica. In the organism model corresponding to this pathology characterized by the early onset of osteoporosis, decorin and biglycan are glycanated to a lesser extent, and it is suggested that this defect is involved in the abnormal gain of periosteum thickness [90]. Equine degenerative suspensory ligament desmitis (DSLD), which affects tendons, ligaments, and other connective tissues and resembles Ehlers–Danlos syndrome, correlates with the accumulation of decorin carrying abnormally glycosylated GAG chains [105]. In this case, chondroitin sulfate replaces the normal dermatan sulfate, and this modification leads to a reduced TGF-β1 binding affinity and the production of antibodies against decorin. There is no change in the level of dermatan sulfate epimerase, the enzyme in charge of the dermatan sulfate synthesis; however, in DSLD, the tendon is associated with high levels of BMP-2, which may enhance proteoglycan production [106,107].

Furthermore, ECM assembly may be influenced by post-translational modifications affecting molecular interactions. These interactions in turn may regulate signaling pathways. The GAG chains of biglycan enhance the binding of calvarial osteoblastic cells from mouse to BMP-4. Consequently, the signaling pathway is more strongly activated, with an increased phosphorylation of Smad 1/5/8 and upregulation of a set of osteoblastic markers such as Cbfa1 (Runx2), osteopontin, bone sialoprotein (BSP) and osteocalcin. Therefore, biglycan plays a role in osteoblast differentiation as a cytokine reservoir thanks to its GAG chains [108]. Biglycan GAGs also play a crucial role on the Erk pathway promoting osteoblast differentiation, as no phosphorylation of Erk is observed when murine pre-osteoblasts are treated with biglycan lacking GAGs [109]. It is conceivable that the age-related evolution of SLRP GAGs is correlated with their balancing function on growth factors as, in juveniles, the osteoblast differentiation process needs to be active and fully functional [108]. Surprisingly, a study showed that although all forms of biglycan positively affect BMP-2 signaling in C2C12 myogenic cells, even if non-glycanated forms are more efficient, only the biglycan lacking GAG induces osteogenesis in a rat mandible-deficient model. The studies concluded that the biglycan positive effect on BMP-2 is inhibited by its GAG moieties. However, it is important to highlight that the cells in these studies are not at the same differentiation stages nor the same cell lines, but a role of GAGs dependent on the type of BMP cannot be excluded [108,110,111].

## 6. Other SLRP Post-Translational Events in Skeletal Tissues

### 6.1. Sulfation

Sulfated tyrosines were identified in fibromodulin, osteomodulin, lumican, and opticin [38,112]. The tyrosine sulfate domain of SLRPs is interacting with proteins, mimicking heparin via the highly negative charge produced in combination with acidic amino acids. Fibromodulin and osteomodulin were shown to bind heparin-binding protein through this specific domain, and the affinity varies via the sulfated tyrosine residues number and their position. When present on the fibromodulin N-terminal domain, it attracts the MMP-13 protease and guides the cleavage of the domain [113,114]. This specific domain is involved in the fibrillogenesis process. Fibromodulin can bind to collagen type I by both its LRR and its N-terminal tyrosine sulfated domain, which allows it to interact simultaneously with two collagen molecules. That feature may be useful in the assembly and networking of the ECM. The sulfated domain affects the collagen fibril formation and induces a shortened lag phase, thus affecting the arrangement of collagen molecules into highly organized fibrils structures [115].

### 6.2. SLRP Degradation and Cleavage in Skeletal Tissues

In addition to their glycosylation and glycanation, SLRPs degradation interferes with their biological functions and adds a level of complexity to their network. In this part, we will discuss the degradation mechanisms of SLRPs and the impact on their biological functions.

Several MMPs have been recognized to cleave SLRP members in vitro. MMP-2, MMP-3, and MMP-7 can cleave the human recombinant decorin. Interestingly, MMP-2 and MMP-3 show decreased efficiency when decorin is lacking its GAG. The consequences of substrate affinity variation with respect to post-translational modification combined with the increase of non-glycanated forms through aging remains unclear [116]. Membrane-type matrix metalloproteinase-1 (MT1-MMP) can induce cleavage of the human recombinant lumican [117]. Fibromodulin is cleaved in the N-terminal region containing sulfated tyrosine when cartilage degradation is induced. MMP-13 can cleave fibromodulin in the region interacting with collagen fibrils [113,118]. MMP-13 is also able to cleave biglycan, opticin, and to a lesser extent, lumican and decorin. In the latter cases, the degradation products are of low abundance despite proven direct interaction between MMP-13 and decorin [119,120,121]. In human cartilage, MMP-1-2-3-7-8-9 were shown to be part of opticin catabolism with different proteolytic efficiencies, with MMP-2 and MMP-7 being the most efficient [122]. MMP-9 and MMP-12 can cleave biglycan, and the fragments produced appear to be relevant neo-epitopes associated with dysregulated ECM remodeling pathogenesis. Indeed, the biglycan fragments were increased in ex vivo degraded cartilage explants and in the serum of a rheumatoid arthritis (RA) rat model [123]. In addition to the MMPs, ADAMTS-4, ADAMTS-5, and granzyme B can be mentioned as efficient enzymes that cleave some SLRPs, as it was proven for biglycan, decorin, and opticin [122,124,125,126]. Chondroadherin can be digested by the serine protease HTRA1 in degenerated human intervertebral discs [127].

The various locations of the cleavage sites lead to numerous possibilities of SLRP fragments to be produced (Table 3). For instance, biglycan is processed in its N-terminal region [88,128] within its core, namely in its fifth LRR by ADAMTS-4 and ADAMTS-5 [124] and in its C-terminal region by MMPs [119]. The biological importance of this large variety of fragments remains an under-examined field and needs further research.

Corroborating the in vitro data, there is evidence that SLRPs are degraded in vivo. During the aging process, decorin and biglycan undergo degradation in bovine tendons [130]. Degraded biglycan, lumican, keratocan, and opticin fragments were found in human articular cartilage, while decorin and fibromodulin fragments were found in remodeling tissues from ovine intervertebral discs after a lesion [119,120,131]. The team of Zhen et al. *(2008)* [132] characterized several MMPs and ADAMTS that carry out the proteolysis of SLRPs; among them were biglycan, decorin, fibromodulin, osteoglycin, and PRELP. They showed that in human cartilage, biglycan, fibromodulin, and PRELP are cleaved by MMP-2, MMP-3, MMP-8, MMP-9, MMP-12, MMP-13, ADAMTS-4, and ADAMTS-5. Decorin can be digested by the same proteases except for MMP-9, lumican by MMP-12 and ADAMTS-4, and osteoglycin by MMP-2, MMP-8, and ADAMTS-4 [132]. The processing of SLRPs occurs in human knee, hip articular cartilage, and meniscus, as it was shown for decorin, biglycan, lumican, and keratocan. This fragmentation process is increased in tissues undergoing degradation, and there is a slight increase related with the aging process but not in every tissue. However, fewer fragments were found in tissues for fibromodulin. Interestingly, but not unexpectedly, fragments observed after in vitro cleavage of biglycan and decorin by MMP-13 correspond to the fragments characterized in vivo, in contrast to the fibromodulin fragments. Yet, it must be highlighted that a majority of the in vivo, so-called naturally occurring fragments do not correlate with fragments generated in vitro. This suggests that besides all the enzymes already identified to cleave the SLRPs, additional unknown enzymes may be involved in their degradation [119,133,134].

This phenomenon may lead to the alteration of ECM homeostasis and its biomechanical properties, and hence damage skeletal tissues over time [119,124,133,135,136]. An increased proteolysis of chondroadherin has also been observed in the scoliotic disc of some adolescent patients and in adult degenerative discs when compared to normal discs. The fragmentation of chondroadherin is also characteristic of the disease, the cleavage site-specific for disc degeneration is represented in Table 2, making the chondroadherin fragment an efficient biomarker [127,137]. In addition, other SLRPs present enhanced fragmentation patterns in pathological human and canine intervertebral discs [138,139,140].

Interestingly, the SLRP fragment pattern has been characterized in serum of osteoarthritic (OA) and RA patients and in the serum of animals with experimentally induced OA. This observation indicates a relationship between these pathologies and the SLRP degradation. The fragmentation pattern is more than a global OA feature; it is also specific to the SLRP member and the joint localization. For example, more cleavage products are detected in OA hip than in OA knee articular cartilage for decorin, biglycan, lumican, and keratocan [133,136,141]. The extent of fibromodulin and opticin degradation by MMP-13 is correlated with the severity of the cartilage damage [113,119,120,142]. Knowing that almost all the members of the SLRP family are involved in collagen interaction as previously reviewed by Chen and Birk, 2013 [6], and that they have a protective function on collagen fibrils, their degradation could lead to the exposure of the MMP-13 cleavage site on the collagen, indicating a predisposition for the initiation of cartilage damage [113,118,119,120]. Consolidating this hypothesis, it was demonstrated that the maximal biglycan processing in the medial meniscus outer zone is concomitant with collagenolysis [126,143]. Moreover, treatment with RS 110–2481, an MMP-13 inhibitor, prevents not only SLRP degradation but also collagenolysis [119,144]. The loss of SLRPs weakens the cartilage’s mechanical properties [119,136].

It appears that SLRP fragments are of interest to unravel the mechanism of OA, and some could be specifically beneficial to study. High levels of biglycan were found in synovial fluid, which is located in the joint cavities of OA and RA patients [145,146]. Treatments with soluble biglycan were reported to induce an inflammatory response in human chondrocytes through TLR-4 and NF-κB activation, enhancing the catabolic response in cartilage explants depending on their OA stage [146,147]. It was also demonstrated that cartilage neo-angiogenesis associated with inflammation [148] is related to the degradation of opticin, which is an inhibitor of angiogenesis, by regulating the adhesiveness of endothelial cells. In OA cartilage, opticin is a substrate for several proteases, and particularly MMP-7 [120,122,149].

The cleavage of SLRPs also impacts the accumulation of growth factors in the ECM. SLRPs are known to bind several growth factors, such as TGF-β, FGF, and BMP, and block their biological activity [150]. Direct evidence of active TGF-β1 being released from decorin and biglycan upon cleavage by granzyme B, a protease that accumulates in the extracellular space during inflammation, was demonstrated. TGF-β1 was also released from decorin after proteolysis by MMP-2, MMP-3, or MM-7. Biglycan, asporin, and fibromodulin were also found to bind TGF-β, giving them the possibility to release it when cleaved [151,152]. Moreover, in SLRP knockout mice, there is an excessive activation of TGF-β1 signaling, leading to an impaired control on osteoprogenitor cells and chondrogenesis. These data suggest a mechanism by which the modulation of the bioavailability of cytokines such as TGF-β1 can correlate to the development or even the initiation of OA [8,116,125,153].

### 6.3. SLRP Intracellular Degradation Pathways

The intracellular catabolism requires the lysosomal system, and SLRPs can accumulate within cells when lysosomes are inhibited [129,154,155,156,157,158]. This mechanism appears to be partially competitive between SLRPs. The internalizing process is receptor-mediated, and the core protein involving the LRR structure is the ligand for the putative 51kDa and 26 kDa human receptors of endocytosis located at the plasma membrane and in the endosomal compartment. Furthermore, the presence of GAG modification adds a supplemental layer of control, as its presence negatively affects the uptake [159,160,161,162,163]. Interactions between SLRPs and other ECM proteins also interfere with the endocytosis, as only free SLRPs are degraded through the internal pathway [164]. Additional human receptors possibly involved in endocytosis were later characterized in a different cell type for decorin or biglycan, suggesting redundancy in receptors involved in SLRP endocytosis. The putative receptors encompass the first discovered 51 kDa and 26 kDa [159]; a 110 kDa receptor [165]; the IGFR [166], and the class A scavenger receptor [167]. In addition, for decorin, it was suggested that several endocytic pathways can be used such as the clathrin-dependant pathway and the Tfr/recycling pathway. It also appears that endocytosis is dependent on the signaling role of EGFR, the PI-3 kinase signaling, and the lipid rafts [168].

## 7. Conclusions

There is a great variability in codon usage among the SLRP coding sequences. This may impact the structure and stability of these proteoglycans. According to their codon usage, it appears that SLRPs split into two main clusters. However, it is currently unclear whether this variability in codon usage plays a functional role at all. Further investigations are required to shed light on the causal mechanisms or chain of events by which codon usage may affect the differential SLRP translation rate and how codon usage is associated with pathological conditions. In summary, current assumptions, which are not necessarily mutually exclusive, are that rare codon usage clusters in the SLRP sequences are required to increase the time available to allow for proper folding or for adequate in vivo co-translational chemical tagging on specific amino acid residues for post-translational purposes, or to cause differential sensitivity to enzymatic tRNA modifications that occur under stressed conditions.

**Figure 4:**
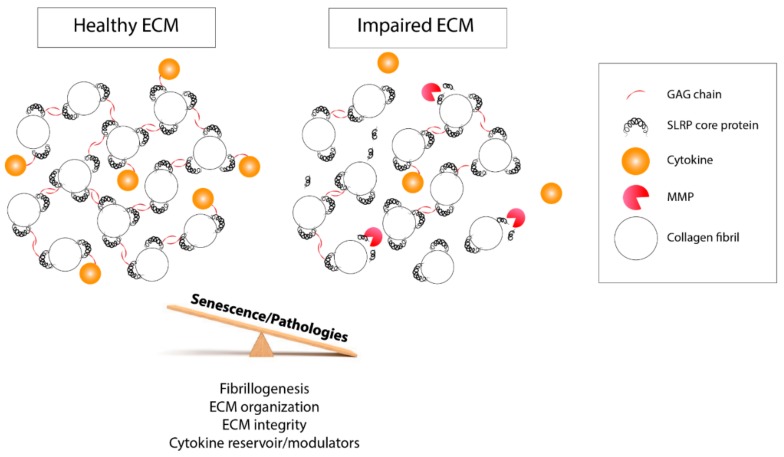
Schematic representation of the biological functions of post-translational modifications impacted by aging and pathology development. On the left panel, GAG side chains and the tyrosine sulfate-rich domain of SLRPs help to maintain the ECM biomechanical properties and the fibrillogenesis and organize the collagen fibrils. They regulate the cell response and their homeostasis through their interaction with cytokines and modulate their accessibility. On the right panel, following an imbalanced control of the post-translational events and MMP-driven degradation, the ECM biological properties are disturbed, which impedes the bioavaibility of cytokines that are free to leave the ECM. This latter case can be prompted by a disease condition or the aging process.

Similarly, there is great variability of glycosylation, GAG, and sulfotyrosine patterns, as well in the SLRP fragments detected. All are subject to changes according to the related tissue, age, and pathological conditions. These heterogeneous post-translational events can contribute to various structural and biological functions of SLRPs. Accordingly, one can define SLRPs as highly versatile components of the ECM. The presence of GAGs and sulfated tyrosine domain conditions their interactions with collagens and ECM proteins, and it can be considered as a factor in ECM assembly and integrity. They are also modulating cytokine activity and availability by sequestering them and are bringing a higher level of subtlety to the cellular response (Figure 4). Moreover, the presence of distinct and precise GAG motifs is involved in specific functions in different cellular contexts [169].

Concerning the SLRP catabolism, it plays a role in cell responses as well by regulating the bioavailability of the molecules under the control of SLRPs. SLRPs themselves can lose their functions and properties under this process. The catabolism can be affected by aging and impaired by pathologies such as Ehlers–Danlos syndrome, OA, RA, or Kashin–Beck disease [170]. SLRPs form a protective coat protecting the ECM from collagenolysis induced by MMPs. Interestingly, alteration of the ECM content appears to be an early step in the progression of a pathology disrupting the ECM properties and functions and later deregulates the cell homeostasis (Figure 4). SLRP fragments can be soluble and circulate in biological fluid, where they might trigger a biological response in other tissues. This means that SLRPs could be actors in aging-related musculoskeletal and rheumatic diseases and could be therapeutic targets. They could also be potential soluble biomarkers that are useful for the diagnosis and prognosis of disease progression as well as predictive of therapeutic response. Indeed, numerous SLRP fragments were identified through mass spectrometry to have the potential to be unique neopeptides characterizing pathologic conditions such as cartilage degradation and OA onset [132,171,172,173,174,175]. In particular, a biglycan neoepitope issued from MMP cleavage measured in rat serum is correlated with pathologies involving imbalanced ECM remodeling [123]. These features could be tracked down to follow the initiation and development of pathologies that are hard to diagnose at an early stage.

In summary, post-translational events encompass a large number of modifications in the SLRP core proteins. They help ECM assembly and are involved in the ECM physicochemical properties and remodeling. They are also required by cells to regulate responses with higher precision. They are valuable fingerprints of the tissues and a characterizing tool that can be used as biomarkers. Here, we focused on the SLRP post-translational events and degradation in skeletal tissues; however, they exert their modulation of biological functions in other tissues as well. Unfortunately, few studies discuss their functions and take them into account when analyzing results. Given the findings summarized in this review, we conclude that their biological functions are too often underestimated, and we hope that future studies will be more careful to consider the impact of post-translational events on biological functions and include them in their design.

## Figures and Tables

**Table 1:** Classification of the SLRP family in five classes; BGN: Biglycan; DCN: Decorin; ASP: Asporin; ECM2: Extracellular Matrix Protein 2; ECMX: Extracellular Matrix Protein X; LUM: Lumican; KTN: Keratocan; FMOD: Fibromodulin; OMD: Osteomodulin; PRELP: Proline/arginine-rich end leucine-rich repeat protein; EPN: Epiphycan; OGN: Osteoglycin; OPTC: Opticin; CHAD: Chondroadherin; NYX: Nyctalopin; TSK: Tsukushi; PODN: Podocan; PODNL: Podocan-like protein. The N-terminal cysteine cluster is a major feature for the classification of this family. The 3D representations are resolved by X-ray diffraction and are publicly available on protein data base repositories such as Protein Data Bank (PDB: https://www.rcsb.org/). The 3D structures can be displayed and viewed with the PyMol software (Schrödinger LCC, version 2.1.1), illustrating the horseshoe shape with lateral asparagines (N shown in red thanks to a home-made Python script). X-Ray crystallographic analysis for the SLRP family members of class III and class IV are not yet available. The different post-translational modifications are listed by each SLRP family member. All information was cross-checked with the UniProt database [28]. LRR: leucine-rich repeat.

Class	N-End Cysteine Motif	3D Representation and PDB ID	Member	GAG Type/Glycosylation	Other	Ref.
**I**	CX_3_CXCX_6_C	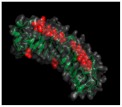 ID: 1XKU	BGN	Chondroitin sulfate Dermatan sulfate N-linked oligosaccharide O-linked oligosaccharide		[29,30,31,32]
		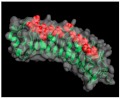 ID: 2FT3	DCN	Chondroitin sulfate Dermatan sulfate N-linked oligosaccharide O-linked oligosaccharide		[30,31,32,33,34]
			ASP	N-linked oligosaccharide O-linked oligosaccharide		[35]
			ECM2	N-linked oligosaccharide No data on potential GAG	ECM2 has a peculiarity in its motif with only 2 conserved cysteines.	[19,31]
			ECMX	No data on potential GAG or glycosylation		[31]
**II**	CX_3_CXCX_9_C		LUM	Keratan sulfate Poly-lactosamine N-linked oligosaccharide	Tyrosine sulfation	[36,37,38]
			KTN			[39]
		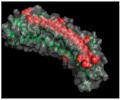 ID: 5MX0	FMOD	Keratan sulfate Poly-lactosamine N-linked oligosaccharide	Tyrosine sulfation Acidic patch	[38,40,41,42]
		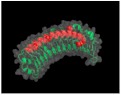 ID: 5YQ5	OMD	Keratan sulfate N-linked oligosaccharide		[38,43,44,45]
			PRELP	N-linked oligosaccharide	Basic patch	[46,47]
**III**	CX_2_CXCX_6_C		EPN	Chondroitin sulfate Dermatan sulfate N-linked oligosaccharide O-linked oligosaccharide	LRRs with only seven repeats Tyrosine sulfation Acidic patch	[30,31]
			OGN	Keratan sulfate Chondroitin sulfate Dermatan sulfate N-linked oligosaccharide	LRRs with only seven repeats Tyrosine sulfation	[31,48,49,50,51,52]
			OPTC	N-linked oligosaccharide O-linked oligosaccharide		[31,53,54]
**IV**	CX_3_CXCX_6-17_C	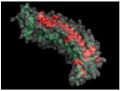 ID: 5MX1	CHAD	Keratan sulfate O-linked oligosaccharide		[31,55]
			NYX TSK	N-linked oligosaccharide		[31]
**V**	CX_3-4_CXCX_9_C		PODN	N-linked oligosaccharide	High number of LRR with 20 repeats Acidic patch	[9,56,57]
			PODNL		High number of LRR with 21 repeats	[58]

**Table 2:** Representation of the biased codon usage patterns of the mature mRNA transcripts among Cluster 1 and Cluster 2 of the SLRP family. Over-represented codons are colored in green and marked by a ‘+’; under-represented codons are colored in red and marked by a ‘–’.

		Cluster 1	Cluster 2
**Leucine**	CUG	+	−
	CUC	+	−
	CUU	−	+
	CUA	−	+
	UUG	−	+
	UUA	−	+
**Glutamate**	GAG	+	−
	GAA	−	+
**Glutamine**	CAG	+	−
	CAA	−	+
**Lysine**	AAG	+	−
	AAA	−	+
**Aspartate**	GAC	+	−
	GAU	−	+
**Asparagine**	AAC	+	−
	AAU	−	+
**Cysteine**	UGC	+	−
	UGU	−	+

**Table 3:** Specific cleavage sites among the SLRP family in the skeletal tissues.

SLRP	Species	Cleavage Site	Protease	Technique	In Vivo Data	Ref
Decorin	Human					
		S241-L242	MMP2	N-terminal sequencing	-	[116]
		S241-L242	MMP3	N-terminal sequencing	-	
		D31-A32	MMP7	N-terminal sequencing	-	
		E274-L273	MMP7	N-terminal sequencing	-	
		S240-L241	MMP-13	N-terminal sequencing	Comparison with WB on cartilage	[119]
	Bovine					
		M200-K201	-	N-terminal sequencing	Extracted from fresh matrix tendon	[129]
		A209-D210	-			
		Q218-G219	-		Extracted from medium of cultured tendon	
Biglycan	Human					
		G177-V178	MMP-13	N-terminal sequencing	Comparison with WB on cartilage	[119]
	Bovine					
		N187-C188	ADAMTS-4 ADAMTS-5	N-terminal sequencing	Comparison with WB on cartilage	[124]
Fibromodulin	Human					
		Y63-T64	MMP-13	Data not shown	Data not shown	[113]
	Bovine					
		Y63-A64	MMP-13	Mass spectrometry	Extracted from cartilage explant	[113]
Opticin	Human					
		T87-S88	MMP-2 MMP-7	N-terminal sequencing	Comparison with WB on cartilage	[122]
		E443-L444	MMP-2 MMP-7			
		G114-L115	MMP-2 MMP-7	Prediction from [120]		
		A20-S21	MMP-7	N-terminal sequencing		
		E32-Q33	MMP-7			
	Bovine					
		G104-L105	MMP-13	N-terminal sequencing	Comparison with WB on human cartilage; IHC on human cartilage and synovial membrane	[120]
		P109-A110	MMP-13			
Chondroadherin	Human					
		I80-Y81	HTRA1	Mass spectrometry	Comparison with WB on discs tissue	[127]

## Supplementary Materials

The supplementary materials are available online at https://www.mdpi.com/2218-273X/10/1/80/s1.

## References

[1] Hynes R.O., Naba A. (2012). Overview of the matrisome-An inventory of extracellular matrix constituents and functions. Cold Spring Harb. Perspect. Biol..

[2] Bonnans C., Chou J., Werb Z. (2014). Remodelling the extracellular matrix in development and disease. Nat. Rev. Mol. Cell Boil..

[3] Naba A., Clauser K.R., Ding H., Whittaker C.A., Carr S.A., Hynes R.O. (2016). The extracellular matrix: Tools and insights for the “omics” era. Matrix Biol..

[4] Kalamajski S., Oldberg A. (2010). The role of small leucine-rich proteoglycans in collagen fibrillogenesis. Matrix Boil..

[5] Nikitovic A., Aggelidakis J., Young M.F., Iozzo R.V., Karamanos N.K., Tzanakakis G.N. (2012). The Biology of Small Leucine-rich Proteoglycans in Bone Pathophysiology. J. Boil. Chem..

[6] Chen S., Birk D.E. (2013). The regulatory roles of small leucine-rich proteoglycans in extracellular matrix assembly. FEBS J..

[7] Chen X.-D., Fisher L.W., Robey P.G., Young M.F. (2004). The small leucine-rich proteoglycan biglycan modulates BMP-4-induced osteoblast differentiation. FASEB J..

[8] Bi Y., Stuelten C.H., Kilts T., Wadhwa S., Iozzo R.V., Robey P.G., Chen X.-D., Young M.F. (2005). Extracellular Matrix Proteoglycans Control the Fate of Bone Marrow Stromal Cells. J. Boil. Chem..

[9] Schaefer L., Iozzo R. (2008). V Biological Functions of the Small Leucine-rich Proteoglycans: From Genetics to Signal Transduction. J. Biol. Chem..

[10] Kram V., Kilts T.M., Bhattacharyya N., Li L., Young M.F. (2017). Small leucine rich proteoglycans, a novel link to osteoclastogenesis. Sci. Rep..

[11] Marín M. (2008). Folding at the rhythm of the rare codon beat. Biotechnol. J..

[12] Plotkin J.B., Kudla G. (2011). Synonymous but not the same: The causes and consequences of codon bias. Nat. Rev. Genet..

[13] Sherman M.Y., Qian S.-B. (2013). Less is more: Improving proteostasis by translation slow down. Trends Biochem. Sci..

[14] Brule C.E., Grayhack E.J. (2017). Synonymous Codons: Choose Wisely for Expression. Trends Genet..

[15] Chan C., Pham P., Dedon P.C., Begley T.J. (2018). Lifestyle modifications: Coordinating the tRNA epitranscriptome with codon bias to adapt translation during stress responses. Genome Boil..

[16] Duan G., Walther D. (2015). The Roles of Post-translational Modifications in the Context of Protein Interaction Networks. PLoS Comput. Boil..

[17] Schaefer L., Schaefer R.M. (2010). Proteoglycans: From structural compounds to signaling molecules. Cell Tissue Res..

[18] Iozzo R.V., Goldoni S., Berendsen A.D., Young M.F. (2011). Small Leucine-Rich Proteoglycans. The Extracellular Matrix: An Overview.

[19] McEwan P.A., Scott P.G., Bishop P.N., Bella J. (2006). Structural correlations in the family of small leucine-rich repeat proteins and proteoglycans. J. Struct. Boil..

[20] Kobe B. (2001). The leucine-rich repeat as a protein recognition motif. Curr. Opin. Struct. Boil..

[21] Chen S., Sun M., Iozzo R.V., Kao W.W.-Y., Birk D.E. (2013). Intracellularly-retained decorin lacking the C-terminal ear repeat causes ER stress: A cell-based etiological mechanism for congenital stromal corneal dystrophy. Am. J. Pathol..

[22] Prydz K., Dalen K.T. (2000). Synthesis and sorting of proteoglycans. J. Cell Sci..

[23] Funderburgh J.L. (2002). Keratan Sulfate Biosynthesis. IUBMB Life.

[24] Fuller M., Meikle P.J., Hopwood J.J. (2004). Glycosaminoglycan degradation fragments in mucopolysaccharidosis I. Glycobiology.

[25] Ernst S., Langer R., Cooney C.L., Sasisekharan R. (1995). Enzymatic Degradation of GlycosaminogIycans. Crit. Rev. Biochem. Mol. Boil..

[26] Krishnan P., Hocking A.M., Scholtz J.M., Pace C.N., Holik K.K., McQuillan D.J. (1999). Distinct secondary structures of the leucine-rich repeat proteoglycans decorin and biglycan. Glycosylation-dependent conformational stability. J. Boil. Chem..

[27] Seo N.-S., Hocking A.M., Höök M., McQuillan D.J. (2005). Decorin Core Protein Secretion Is Regulated byN-Linked Oligosaccharide and Glycosaminoglycan Additions. J. Boil. Chem..

[28] Bateman A. (2019). UniProt: A worldwide hub of protein knowledge. Nucleic Acids Res..

[29] Fisher L.W., Termine J.D., Young M.F. (1989). Deduced protein sequence of bone small proteoglycan I (biglycan) shows homology with proteoglycan II (decorin) and several nonconnective tissue proteins in a variety of species. J. Boil. Chem..

[30] Johnson H.J., Rosenberg L., Choi H.U., Garza S., Höök M., Neame P.J. (1997). Characterization of epiphycan, a small proteoglycan with a leucine-rich repeat core protein. J. Boil. Chem..

[31] Iozzo R.V., Schaefer L. (2015). Proteoglycan form and function: A comprehensive nomenclature of proteoglycans. Matrix Boil..

[32] Roughley P.J., White R.J. (1989). Dermatan sulphate proteoglycans of human articular cartilage. The properties of dermatan sulphate proteoglycans I and II. Biochem. J..

[33] Krusius T., Ruoslahti E. (1986). Primary structure of an extracellular matrix proteoglycan core protein deduced from cloned cDNA. Proc. Natl. Acad. Sci. USA.

[34] Klein J.A., Meng L., Zaia J. (2018). Deep sequencing of complex proteoglycans: A novel strategy for high coverage and sitespecific identification of glycosaminoglycanlinked peptides. Mol. Cell. Proteom..

[35] Lorenzo P., Aspberg A., Önnerfjord P., Bayliss M.T., Neame P.J., Heinegård D. (2001). Identification and Characterization of Asporin. J. Boil. Chem..

[36] Blochberger T.C., Vergnes J.P., Hempel J., Hassell J.R. (1992). cDNA to chick lumican (corneal keratan sulfate proteoglycan) reveals homology to the small interstitial proteoglycan gene family and expression in muscle and intestine. J. Boil. Chem..

[37] Cornuet P.K., Blochberger T.C., Hassell J.R. (1994). Molecular polymorphism of lumican during corneal development. Investig. Ophthalmol. Vis. Sci..

[38] Önnerfjord P., Heathfield T.F., Heinegård D. (2004). Identification of Tyrosine Sulfation in Extracellular Leucine-rich Repeat Proteins Using Mass Spectrometry. J. Biol. Chem..

[39] Corpuz L.M., Funderburgh J.L., Funderburgh M.L., Bottomley G.S., Prakash S., Conrad G.W. (1996). Molecular cloning and tissue distribution of keratocan. Bovine corneal keratan sulfate proteoglycan 37A. J. Boil. Chem..

[40] Oldberg A., Antonsson P., Lindblom K., Heinegård D. (1989). A collagen-binding 59-kd protein (fibromodulin) is structurally related to the small interstitial proteoglycans PG-S1 and PG-S2 (decorin). EMBO J..

[41] Antonsson P., Heinegird D., Oldberg A. (1991). Posttranslational Modifications of Fibromodulin. J. Biol. Chem..

[42] Plaas A.H., Wong-Palms S. (1993). Biosynthetic mechanisms for the addition of polylactosamine to chondrocyte fibromodulin. J. Boil. Chem..

[43] Wendel M., Sommarin Y., Heinegård D. (1995). Characterization of osteoadherin—a novel, cell binding keratan sulfate proteoglycan from bone. Acta Orthop. Scand..

[44] Wendel M., Sommarin Y., Heinegård D. (1998). Bone Matrix Proteins: Isolation and Characterization of a Novel Cell-binding Keratan Sulfate Proteoglycan (Osteoadherin) from Bovine Bone. J. Cell Boil..

[45] Sommarin Y. (1998). Osteoadherin, a Cell-binding Keratan Sulfate Proteoglycan in Bone, Belongs to the Family of Leucine-rich Repeat Proteins of the Extracellular Matrix. J. Boil. Chem..

[46] Bengtsson E., Neame P.J., Heinegård D., Sommarin Y. (1995). The Primary Structure of a Basic Leucine-rich Repeat Protein, PRELP, Found in Connective Tissues. J. Biol. Chem..

[47] Bengtsson E., Aspberg A., Heinegãrd D., Sommarin Y., Spillmann D. (2000). The Amino-terminal Part of PRELP Binds to Heparin and Heparan Sulfate. J. Boil. Chem..

[48] Bentz H., Nathan R.M., Rosen D.M., Armstrong R.M., Thompson A.Y., Segarini P.R., Mathews M.C., Dasch J.R., Piez K.A., Seyedin S.M. (1989). Purification and characterization of a unique osteoinductive factor from bovine bone. J. Boil. Chem..

[49] Funderburgh J.L., Corpuz L.M., Roth M.R., Funderburgh M.L., Tasheva E.S., Conrad G.W. (1997). Mimecan, the 25-kDa corneal keratan sulfate proteoglycan, is a product of the gene producing osteoglycin. J. Boil. Chem..

[50] Kampmann A., Fernández B., Deindl E., Kubin T., Pipp F., Eitenmüller I., Hoefer I.E., Schaper W., Zimmermann R. (2009). The proteoglycan osteoglycin/mimecan is correlated with arteriogenesis. Mol. Cell. Biochem..

[51] Rienks M., Papageorgiou A., Wouters K., Verhesen W., van Leeuwen R., Carai P., Summer G., Westermann D., Heymans S. (2017). A novel 72-kDa leukocyte-derived osteoglycin enhances the activation of toll-like receptor 4 and exacerbates cardiac inflammation during viral myocarditis. Cell. Mol. Life Sci..

[52] Madisen L., Neubauer M., Plowman G., Rosen D., Segarini P., Dasch J., Thompson A., Ziman J., Bentz H., Purchio A. (1990). Molecular Cloning of a Novel Bone-Forming Compound: Osteoinductive Factor. DNA Cell Boil..

[53] Reardon A.J., Le Goff M., Briggs M.D., McLeod D., Sheehan J.K., Thornton D.J., Bishop P.N. (2000). Identification in vitreous and molecular cloning of opticin, a novel member of the family of leucine-rich repeat proteins of the extracellular matrix. J. Boil. Chem..

[54] Hobby P., Wyatt M.K., Gan W., Bernstein S., Tomarev S., Slingsby C., Wistow G. (2000). Cloning, modeling, and chromosomal localization for a small leucine-rich repeat proteoglycan (SLRP) family member expressed in human eye. Mol. Vis..

[55] Neame P.J., Sommarin Y., Boynton R.E., Heinegård D. (1994). The structure of a 38-kDa leucine-rich protein (chondroadherin) isolated from bovine cartilage. J. Boil. Chem..

[56] Shimizu-Hirota R., Sasamura H., Kuroda M., Kobayashi E., Saruta T. (2004). Functional characterization of podocan, a member of a new class in the small leucine-rich repeat protein family. FEBS Lett..

[57] Ross M.D., Bruggeman L.A., Hanss B., Marras D., Klotman M.E., Sunamoto M., Klotman P.E. (2003). Podocan, a Novel Small Leucine-rich Repeat Protein Expressed in the Sclerotic Glomerular Lesion of Experimental HIV-associated Nephropathy. J. Boil. Chem..

[58] Mochida Y., Kaku M., Yoshida K., Katafuchi M., Atsawasuwan P., Yamauchi M. (2011). Podocan-like protein: A novel small leucine-rich repeat matrix protein in bone. Biochem. Biophys. Res. Commun..

[59] Endres L., Dedon P.C., Begley T.J. (2015). Codon-biased translation can be regulated by wobble-base tRNA modification systems during cellular stress responses. RNA Biol..

[60] Rapino F., Delaunay S., Zhou Z., Chariot A., Close P. (2017). tRNA Modification: Is Cancer Having a Wobble?. Trends Cancer.

[61] Hou Y.-M., Gamper H., Yang W. (2015). Post-transcriptional modifications to tRNA--a response to the genetic code degeneracy. RNA.

[62] Rapino F., Delaunay S., Rambow F., Zhou Z., Tharun L., De Tullio P., Sin O., Shostak K., Schmitz S., Piepers J. (2018). Codon-specific translation reprogramming promotes resistance to targeted therapy. Nat..

[63] James G., Witten D., Hastie T., Tibshirani R. (2013). An Introduction to Statistical Learning.

[64] Raspanti M., Viola M., Forlino A., Tenni R., Gruppi C., Tira M.E. (2008). Glycosaminoglycans show a specific periodic interaction with type I collagen fibrils. J. Struct. Boil..

[65] Tatara Y., Kakizaki I., Suto S., Ishioka H., Negishi M., Endo M. (2015). Chondroitin sulfate cluster of epiphycan from salmon nasal cartilage defines binding specificity to collagens. Glycobiology.

[66] Lewis P.N., Pinali C., Young R.D., Meek K.M., Quantock A.J., Knupp C. (2010). Structural Interactions between Collagen and Proteoglycans Are Elucidated by Three-Dimensional Electron Tomography of Bovine Cornea. Structure.

[67] Parfitt G.J., Pinali C., Young R.D., Quantock A.J., Knupp C. (2010). Three-dimensional reconstruction of collagen–proteoglycan interactions in the mouse corneal stroma by electron tomography. J. Struct. Boil..

[68] Stamov D.R., Müller A., Wegrowski Y., Brezillon S., Franz C.M., Müller A. (2013). Quantitative analysis of type I collagen fibril regulation by lumican and decorin using AFM. J. Struct. Boil..

[69] Scott J.E. (1995). Extracellular matrix, supramolecular organisation and shape. J. Anat..

[70] Scott J.E., Thomlinson A.M. (1998). The structure of interfibrillar proteoglycan bridges (‘shape modules’) in extracellular matrix of fibrous connective tissues and their stability in various chemical environments. J. Anat..

[71] Lujan T.J., Underwood C.J., Henninger H.B., Thompson B.M., Weiss J.A. (2007). Effect of dermatan sulfate glycosaminoglycans on the quasi-static material properties of the human medial collateral ligament. J. Orthop. Res..

[72] Seidler D.G., Faiyaz-Ul-Haque M., Hansen U., Yip G.W., Zaidi S.H.E., Teebi A.S., Kiesel L., Götte M. (2006). Defective glycosylation of decorin and biglycan, altered collagen structure, and abnormal phenotype of the skin fibroblasts of an Ehlers–Danlos syndrome patient carrying the novel Arg270Cys substitution in galactosyltransferase I (β4GalT-7). J. Mol. Med..

[73] Rühland C., Schönherr E., Robenek H., Hansen U., Iozzo R.V., Bruckner P., Seidler D.G. (2007). The glycosaminoglycan chain of decorin plays an important role in collagen fibril formation at the early stages of fibrillogenesis. FEBS J..

[74] Malfait F., Kariminejad A., Van Damme T., Gauche C., Syx D., Merhi-Soussi F., Gulberti S., Symoens S., Vanhauwaert S., Willaert A. (2013). Defective initiation of glycosaminoglycan synthesis due to B3GALT6 mutations causes a pleiotropic Ehlers-Danlos-syndrome-like connective tissue disorder. Am. J. Hum. Genet..

[75] Moffatt P., Geng Y., Lamplugh L., Nanci A., Roughley P.J. (2017). Absence of the dermatan sulfate chain of decorin does not affect mouse development. J. Negat. Results Biomed..

[76] Reese S.P., Underwood C.J., Weiss J.A. (2013). Effects of decorin proteoglycan on fibrillogenesis, ultrastructure, and mechanics of type I collagen gels. Matrix Boil..

[77] Scott J.E. (2003). Elasticity in extracellular matrix ‘shape modules’ of tendon, cartilage, etc. A sliding proteoglycan-filament model. J. Physiol..

[78] Redaelli A., Vesentini S., Soncini M., Vena P., Mantero S., Montevecchi F. (2003). Possible role of decorin glycosaminoglycans in fibril to fibril force transfer in relative mature tendons—A computational study from molecular to microstructural level. J. Biomech..

[79] Vesentini S., Redaelli A., Montevecchi F.M. (2005). Estimation of the binding force of the collagen molecule-decorin core protein complex in collagen fibril. J. Biomech..

[80] Liao J., Vesely I. (2007). Skewness angle of interfibrillar proteoglycans increases with applied load on mitral valve chordae tendineae. J. Biomech..

[81] Lujan T.J., Underwood C.J., Jacobs N.T., Weiss J.A. (2009). Contribution of glycosaminoglycans to viscoelastic tensile behavior of human ligament. J. Appl. Physiol..

[82] Fessel G., Snedeker J.G. (2011). Equivalent stiffness after glycosaminoglycan depletion in tendon—An ultra-structural finite element model and corresponding experiments. J. Theor. Boil..

[83] Svensson R.B., Hassenkam T., Hansen P., Kjaer M., Magnusson S.P. (2011). Tensile Force Transmission in Human Patellar Tendon Fascicles Is Not Mediated by Glycosaminoglycans. Connect. Tissue Res..

[84] Choi R.K., Smith M.M., Martin J.H., Clarke J.L., Dart A.J., Little C.B., Clarke E.C. (2016). Chondroitin sulphate glycosaminoglycans contribute to widespread inferior biomechanics in tendon after focal injury. J. Biomech..

[85] Rigozzi S., Müller R., Stemmer A., Snedeker J. (2013). Tendon glycosaminoglycan proteoglycan sidechains promote collagen fibril sliding—AFM observations at the nanoscale. J. Biomech..

[86] Tatara Y., Suto S., Itoh K. (2017). Novel roles of glycosaminoglycans in the degradation of type I collagen by cathepsin K. Glycobiology.

[87] Johnstone B., Markopoulos M., Neame P., Caterson B. (1993). Identification and characterization of glycanated and non-glycanated forms of biglycan and decorin in the human intervertebral disc. Biochem. J..

[88] Roughley P.J., White R.J., Magny M.C., Liu J., Pearce R.H., Mort J.S. (1993). Non-proteoglycan forms of biglycan increase with age in human articular cartilage. Biochem. J..

[89] Waddington R., Hall R., Embery G., Lloyd D. (2003). Changing profiles of proteoglycans in the transition of predentine to dentine. Matrix Boil..

[90] Chan W.L., Steiner M., Witkos T., Egerer J., Busse B., Mizumoto S., Pestka J.M., Zhang H., Hausser I., Khayal L.A. (2018). Impaired proteoglycan glycosylation, elevated TGF-β signaling, and abnormal osteoblast differentiation as the basis for bone fragility in a mouse model for gerodermia osteodysplastica. PLoS Genet..

[91] Derwin K.A., Soslowsky L.J., Kimura J.H., Plaas A.H. (2001). Proteoglycans and glycosaminoglycan fine structure in the mouse tail tendon fascicle. J. Orthop. Res..

[92] Roughley P.J., White R.J., Cs-Szabó G., Mort J.S. (1996). Changes with age in the structure of fibromodulin in human articular cartilage. Osteoarthr. Cartil..

[93] Santer V., White R.J., Roughley P.J. (1982). O-linked oligosaccharides of human articular cartilage proteoglycan. BBA Gen. Subj..

[94] Grover J., Chen X.N., Korenberg J.R., Roughley P.J. (1995). The human lumican gene. Organization, chromosomal location, and expression in articular cartilage. J. Boil. Chem..

[95] Melching L.I., Roughley P.J. (1999). Modulation of keratan sulfate synthesis on lumican by the action of cytokines on human articular chondrocytes. Matrix Boil..

[96] Sugars R.V., Olsson M.L., Marchner S., Hultenby K., Wendel M. (2013). The glycosylation profile of osteoadherin alters during endochondral bone formation. Bone.

[97] Waddington R.J., Roberts H.C., Sugars R.V., Schönherr E. (2003). Differential roles for small leucine-rich proteoglycans in bone formation. Eur Cell Mater.

[98] Goldberg M., Rapoport O., Septier D., Palmier K., Hall R., Embery G., Young M., Ameye L. (2003). Proteoglycans in predentin: The last 15 micrometers before mineralization. Connect. Tissue Res..

[99] Ho S.P., Sulyanto R.M., Marshall S.J., Marshall G.W. (2005). The cementum–dentin junction also contains glycosaminoglycans and collagen fibrils. J. Struct. Boil..

[100] Bertassoni L.E., Stankoska K., Swain M.V. (2012). Insights into the structure and composition of the peritubular dentin organic matrix and the lamina limitans. Micron.

[101] Bertassoni L.E., Kury M., Rathsam C., Little C.B., Swain M.V. (2015). The role of proteoglycans in the nanoindentation creep behavior of human dentin. J. Mech. Behav. Biomed. Mater..

[102] Dorvee J.R., Gerkowicz L., Bahmanyar S., Deymier-Black A., Veis A. (2016). Chondroitin sulfate is involved in the hypercalcification of the organic matrix of bovine peritubular dentin. Arch. Oral Biol..

[103] Farina A.P., Vidal C.M.P., Cecchin D., Aguiar T.R., Bedran-Russo A.K. (2019). Structural and biomechanical changes to dentin extracellular matrix following chemical removal of proteoglycans. Odontology.

[104] Gutierrez J., Osses N., Brandan E. (2006). Changes in secreted and cell associated proteoglycan synthesis during conversion of myoblasts to osteoblasts in response to bone morphogenetic protein-2: Role of decorin in cell response to BMP-2. J. Cell Physiol..

[105] Halper J., Kim B., Khan A., Yoon J.H., Mueller P.O.E. (2006). Degenerative suspensory ligament desmitis as a systemic disorder characterized by proteoglycan accumulation. BMC Vet. Res..

[106] Kim B., Yoon J.H., Zhang J., Mueller P.E., Halper J. (2010). Glycan profiling of a defect in decorin glycosylation in equine systemic proteoglycan accumulation, a potential model of progeroid form of Ehlers-Danlos syndrome. Arch. Biochem. Biophys..

[107] Young M., Moshood O., Zhang J., Sarbacher C.A., Mueller P.O.E., Halper J. (2018). Does BMP2 play a role in the pathogenesis of equine degenerative suspensory ligament desmitis?. BMC Res. Notes.

[108] Ye Y., Hu W., Guo F., Zhang W., Wang J., Chen A. (2012). Glycosaminoglycan chains of biglycan promote bone morphogenetic protein-4-induced osteoblast differentiation. Int. J. Mol. Med..

[109] Wang X., Harimoto K., Xie S., Cheng H., Liu J., Wang Z. (2010). Matrix protein biglycan induces osteoblast differentiation through extracellular signal-regulated kinase and Smad pathways. Boil. Pharm. Bull..

[110] Miguez P., Terajima M., Nagaoka H., Mochida Y., Yamauchi M. (2011). Role of Glycosaminoglycans of Biglycan in BMP-2 Signaling. Biochem. Biophys. Res. Commun..

[111] Miguez P., Terajima M., Nagaoka H., Ferreira J., Braswell K., Ko C., Yamauchi M. (2014). Recombinant Biglycan Promotes Bone Morphogenetic Protein-induced Osteogenesis. J. Dent. Res..

[112] Kanan Y., Siefert J.C., Kinter M., Al-Ubaidi M.R. (2014). Complement factor H, vitronectin, and opticin are tyrosine-sulfated proteins of the retinal pigment epithelium. PLoS ONE.

[113] Heathfield T.F., Önnerfjord P., Dahlberg L., Heinegård D. (2004). Cleavage of Fibromodulin in Cartilage Explants Involves Removal of the N-terminal Tyrosine Sulfate-rich Region by Proteolysis at a Site That Is Sensitive to Matrix Metalloproteinase-13. J. Biol. Chem..

[114] Tillgren V., Önnerfjord P., Haglund L., Heinegård D. (2009). The Tyrosine Sulfate-rich Domains of the LRR Proteins Fibromodulin and Osteoadherin Bind Motifs of Basic Clusters in a Variety of Heparin-binding Proteins, Including Bioactive Factors. J. Boil. Chem..

[115] Tillgren V., Mörgelin M., Önnerfjord P., Kalamajski S., Aspberg A. (2016). The Tyrosine Sulfate Domain of Fibromodulin Binds Collagen and Enhances Fibril Formation. J. Boil. Chem..

[116] Imai K., Hiramatsu A., Fukushima D., Pierschbacher M.D., Okada Y. (1997). Degradation of decorin by matrix metalloproteinases: Identification of the cleavage sites, kinetic analyses and transforming growth factor-β1 release. Biochem. J..

[117] Li Y., Aoki T., Mori Y., Ahmad M., Miyamori H., Takino T., Sato H. (2004). Cleavage of Lumican by Membrane-Type Matrix Metalloproteinase-1 Abrogates This Proteoglycan-Mediated Suppression of Tumor Cell Colony Formation in Soft Agar. Cancer Res..

[118] Geng Y., McQuillan D., Roughley P.J. (2006). SLRP interaction can protect collagen fibrils from cleavage by collagenases. Matrix Boil..

[119] Monfort J., Tardif G., Reboul P., Mineau F., Roughley P., Pelletier J.-P., Martel-Pelletier J. (2006). Degradation of small leucine-rich repeat proteoglycans by matrix metalloprotease-13: Identification of a new biglycan cleavage site. Arthritis Res. Ther..

[120] Monfort J., Tardif G., Roughley P., Reboul P., Boileau C., Bishop P., Pelletier J.-P., Martel-Pelletier J. (2008). Identification of opticin, a member of the small leucine-rich repeat proteoglycan family, in human articular tissues: A novel target for MMP-13 in osteoarthritis. Osteoarthr. Cartil..

[121] Zhang L., Yang M., Yang N., Cavey G., Davidson P., Gibson G. (2010). Molecular Interactions of MMP-13 C-Terminal Domain with Chondrocyte Proteins. Connect. Tissue Res..

[122] Tío L., Martel-Pelletier J., Pelletier J.-P., Bishop P.N., Roughley P., Farran A., Benito P., Monfort J. (2014). Characterization of opticin digestion by proteases involved in osteoarthritis development. Jt. Bone Spine.

[123] Genovese F., Barascuk N., Larsen L., Larsen M.R., Nawrocki A., Li Y., Zheng Q., Wang J., Veidal S.S., Leeming D.J. (2013). Biglycan fragmentation in pathologies associated with extracellular matrix remodeling by matrix metalloproteinases. Fibrogenesis Tissue Repair.

[124] Melching L., Fisher W., Lee E., Mort J., Roughley P. (2006). The cleavage of biglycan by aggrecanases. Osteoarthr. Cartil..

[125] Boivin W.A., Shackleford M., Vanden Hoek A., Zhao H., Hackett T.L., Knight D.A., Granville D.J. (2012). Granzyme B Cleaves Decorin, Biglycan and Soluble Betaglycan, Releasing Active Transforming Growth Factor-β1. PLoS ONE.

[126] Fuller E., Little C.B., Melrose J. (2016). Interleukin-1α induces focal degradation of biglycan and tissue degeneration in an in-vitro ovine meniscal model. Exp. Mol. Pathol..

[127] Akhatib B., Onnerfjord P., Gawri R., Ouellet J., Jarzem P., Heinegård D., Mort J., Roughley P., Haglund L. (2013). Chondroadherin Fragmentation Mediated by the Protease HTRA1 Distinguishes Human Intervertebral Disc Degeneration from Normal Aging. J. Boil. Chem..

[128] Scott I.C., Imamura Y., Pappano W.N., Troedel J.M., Recklies A.D., Roughley P.J., Greenspan D.S. (2000). Bone Morphogenetic Protein-1 Processes Probiglycan. J. Boil. Chem..

[129] Samiric T., Ilic M.Z., Handley C.J. (2004). Characterisation of proteoglycans and their catabolic products in tendon and explant cultures of tendon. Matrix Biol..

[130] Rees S.G., Flannery C.R., Little C.B., Hughes C.E., Caterson B., Dent C.M. (2000). Catabolism of aggrecan, decorin and biglycan in tendon. Biochem. J..

[131] Melrose J., Smith S.M., Fuller E.S., Young A.A., Roughley P.J., Dart A., Little C.B. (2007). Biglycan and fibromodulin fragmentation correlates with temporal and spatial annular remodelling in experimentally injured ovine intervertebral discs. Eur. Spine J..

[132] Zhen E.Y., Brittain I.J., Laska D.A., Mitchell P.G., Sumer E.U., Karsdal M.A., Duffin K.L. (2008). Characterization of metalloprotease cleavage products of human articular cartilage. Arthritis Rheum..

[133] Melrose J., Fuller E.S., Roughley P.J., Smith M.M., Kerr B., Hughes C.E., Caterson B., Little C.B. (2008). Fragmentation of decorin, biglycan, lumican and keratocan is elevated in degenerate human meniscus, knee and hip articular cartilages compared with age-matched macroscopically normal and control tissues. Arthritis Res. Ther..

[134] Shu C.C., Flannery C.R., Little C.B., Melrose J. (2019). Catabolism of Fibromodulin in Developmental Rudiment and Pathologic Articular Cartilage Demonstrates Novel Roles for MMP-13 and ADAMTS-4 in C-terminal Processing of SLRPs. Int. J. Mol. Sci..

[135] Cs-Szabo G., Roughley P.J., Plaas A.H.K., Glant T.T. (1995). Large and small proteoglycans of osteoarthritic and rheumatoid articular cartilage. Arthritis Rheum..

[136] Young A.A., Smith M.M., Smith S.M., Cake M.A., Ghosh P., Read R.A., Melrose J., Sonnabend D.H., Roughley P.J., Little C.B. (2005). Regional assessment of articular cartilage gene expression and small proteoglycan metabolism in an animal model of osteoarthritis. Arthritis Res. Ther..

[137] Haglund L., Ouellet J., Roughley P. (2009). Variation in Chondroadherin Abundance and Fragmentation in the Human Scoliotic Disc. Spine.

[138] Brown S., Melrose J., Caterson B., Roughley P., Eisenstein S.M., Roberts S. (2012). A comparative evaluation of the small leucine-rich proteoglycans of pathological human intervertebral discs. Spine J..

[139] Erwin W.M., DeSouza L., Funabashi M., Kawchuk G., Karim M.Z., Kim S., Mädler S., Matta A., Wang X., Mehrkens K.A. (2015). The biological basis of degenerative disc disease: Proteomic and biomechanical analysis of the canine intervertebral disc. Arthritis Res..

[140] Bisson D.G., Lama P., Abduljabbar F., Rosenzweig D.H., Saran N., Ouellet J.A., Haglund L. (2018). Facet joint degeneration in adolescent idiopathic scoliosis. JOR Spine.

[141] Bock H., Michaeli P., Bode C., Schultz W., Kresse H., Herken R., Miosge N. (2001). The small proteoglycans decorin and biglycan in human articular cartilage of late-stage osteoarthritis. Osteoarthr. Cartil..

[142] Goldring M.B., Otero M., Plumb D.A., Dragomir C., Favero M., El Hachem K., Hashimoto R., Roach H.I., Olivotto E., Borzì R.M. (2011). Roles of inflammatory and anabolic cytokines in cartilage metabolism: Signals and multiple effectors converge upon MMP-13 regulation in osteoarthritis. Cells Mater..

[143] Fuller E., Smith M., Little C., Melrose J. (2012). Zonal differences in meniscus matrix turnover and cytokine response. Osteoarthr. Cartil..

[144] Billinghurst R.C., Dahlberg L., Ionescu M., Reiner A., Bourne R., Rorabeck C., Mitchell P., Hambor J., Diekmann O., Tschesche H. (1997). Enhanced cleavage of type II collagen by collagenases in osteoarthritic articular cartilage. J. Clin. Investig..

[145] Frey H., Schroeder N., Manon-Jensen T., Iozzo R.V., Schaefer L. (2013). Biological interplay between proteoglycans and their innate immune receptors in inflammation. FEBS J..

[146] Barreto G., Soininen A., Ylinen P., Sandelin J., Konttinen Y.T., Nordström D.C., Eklund K.K. (2015). Soluble biglycan: A potential mediator of cartilage degradation in osteoarthritis. Arthritis Res..

[147] Avenoso A., D’Ascola A., Scuruchi M., Mandraffino G., Calatroni A., Saitta A., Campo S., Campo G.M. (2018). The proteoglycan biglycan mediates inflammatory response by activating TLR-4 in human chondrocytes: Inhibition by specific siRNA and high polymerized Hyaluronan. Arch. Biochem. Biophys..

[148] Bonnet C.S., Walsh D.A. (2005). Osteoarthritis, angiogenesis and inflammation. Rheumatology.

[149] Le Goff M.M., Sutton M.J., Slevin M., Latif A., Humphries M.J., Bishop P.N. (2012). Opticin exerts its anti-angiogenic activity by regulating extracellular matrix adhesiveness. J. Boil. Chem..

[150] Merline R., Schaefer R.M., Schaefer L. (2009). The matricellular functions of small leucine-rich proteoglycans (SLRPs). J. Cell Commun. Signal..

[151] Hildebrand A., Romaris M., Rasmussen L.M., Heinegård D., Twardzik D.R., Border W.A., Ruoslahti E. (1994). Interaction of the small interstitial proteoglycans biglycan, decorin and fibromodulin with transforming growth factor β. Biochem. J..

[152] Kizawa H., Kou I., Iida A., Sudo A., Miyamoto Y., Fukuda A., Mabuchi A., Kotani A., Kawakami A., Yamamoto S. (2005). An aspartic acid repeat polymorphism in asporin inhibits chondrogenesis and increases susceptibility to osteoarthritis. Nat. Genet..

[153] Embree M.C., Kilts T.M., Ono M., Inkson C.A., Syed-Picard F., Karsdal M.A., Oldberg A., Bi Y., Young M.F. (2010). Biglycan and Fibromodulin Have Essential Roles in Regulating Chondrogenesis and Extracellular Matrix Turnover in Temporomandibular Joint Osteoarthritis. Am. J. Pathol..

[154] Campbell M.A., Handley C.J., Hascall V.C., Campbell R.A., Lowther D.A. (1984). Turnover of proteoglycans in cultures of bovine articular cartilage. Arch. Biochem. Biophys..

[155] Campbell M.A., Winter A.D., Ilic M.Z., Handley C.J. (1996). Catabolism and Loss of Proteoglycans from Cultures of Bovine Collateral Ligament. Arch. Biochem. Biophys..

[156] Winter A.D., Campbell M.A., Robinson H., Handley C.J. (2000). Catabolism of newly synthesized decorin by explant cultures of bovine ligament. Matrix Boil..

[157] Samiric T., Ilic M.Z., Handley C.J. (2004). Large aggregating and small leucine-rich proteoglycans are degraded by different pathways and at different rates in tendon. JBIC J. Boil. Inorg. Chem..

[158] Ilic M.Z., Carter P., Tyndall A., Dudhia J., Handley C.J. (2005). Proteoglycans and catabolic products of proteoglycans present in ligament. Biochem. J..

[159] Hausser H., Hoppe W., Rauch U., Kresse H. (1989). Endocytosis of a small dermatan sulphate proteoglycan. Identification of binding proteins. Biochem. J..

[160] Hausser H. (1991). Binding of heparin and of the small proteoglycan decorin to the same endocytosis receptor proteins leads to different metabolic consequences. J. Cell Boil..

[161] Hausser H., Ober B., Quentin-Hoffmann E., Schmidt B., Kresse H. (1992). Endocytosis of Different Members of the Small Chondroitin/Dermatan Sulfate Proteoglycan Family. J. Biol. Chem..

[162] Hausser H., Schönherr E., Müller M., Liszio C., Bin Z., Fisher L.W., Kresse H. (1998). Receptor-Mediated Endocytosis of Decorin: Involvement of Leucine-Rich Repeat Structures. Arch. Biochem. Biophys..

[163] Hausser H., Kresse H. (1999). Decorin endocytosis: Structural features of heparin and heparan sulphate oligosaccharides interfering with receptor binding and endocytosis. Biochem. J..

[164] Schmidt G., Hausser H., Kresse H. (1990). Extracellular accumulation of small dermatan sulphate proteoglycan II by interference with the secretion-recapture pathway. Biochem. J..

[165] Feugaing D.D.S., Kresse H., Greb R.R., Götte M. (2006). A Novel 110-kDa Receptor Protein is Involved in Endocytic Uptake of Decorin by Human Skin Fibroblasts. Sci. World J..

[166] Schönherr E., Sunderkotter C., Iozzo R., Schaefer L. (2005). Decorin, a Novel Player in the Insulin-like Growth Factor System. J. Boil. Chem..

[167] Santiago-García J., Kodama T., Pitas R.E. (2003). The class A scavenger receptor binds to proteoglycans and mediates adhesion of macrophages to the extracellular matrix. J. Biol. Chem..

[168] Feugaing D., Tammi R., Echtermeyer F., Stenmark H., Kresse H., Smollich M., Schönherr E., Kiesel L., Götte M. (2007). Endocytosis of the dermatan sulfate proteoglycan decorin utilizes multiple pathways and is modulated by epidermal growth factor receptor signaling. Biochimie.

[169] Townley R.A., Bülow H.E. (2018). Deciphering functional glycosaminoglycan motifs in development. Curr. Opin. Struct. Boil..

[170] Wang M., Xue S., Fang Q., Zhang M., He Y., Zhang Y., Lammi M.J., Cao J., Chen J. (2019). Expression and localization of the small proteoglycans decorin and biglycan in articular cartilage of Kashin-Beck disease and rats induced by T-2 toxin and selenium deficiency. Glycoconj. J..

[171] Cillero-Pastor B., Eijkel G.B., Kiss A., Blanco F.J., Heeren R.M.A., Garcia F.J.B. (2013). Matrix-assisted laser desorption ionization-imaging mass spectrometry: A new methodology to study human osteoarthritic cartilage. Arthritis Rheum..

[172] Balakrishnan L., Nirujogi R.S., Ahmad S., Bhattacharjee M., Manda S.S., Renuse S., Kelkar D.S., Subbannayya Y., Raju R., Goel R. (2014). Poteomic analysis of human osteoarthritis synovial fluid. Clin. Proteom..

[173] Peffers M.J., Cillero-Pastor B., Eijkel G.B., Clegg P.D., Heeren R.M. (2014). Matrix assisted laser desorption ionization mass spectrometry imaging identifies markers of ageing and osteoarthritic cartilage. Arthritis Res. Ther..

[174] Peffers M., McDermott B., Clegg P., Riggs C. (2015). Comprehensive protein profiling of synovial fluid in osteoarthritis following protein equalization. Osteoarthr. Cartil..

[175] Peffers M.J., Thornton D.J., Clegg P.D. (2016). Characterization of neopeptides in equine articular cartilage degradation. J. Orthop. Res..

