# Supplementary material for: From Translation to Protein Degradation as Mechanisms for Regulating Biological Functions: A Review on the SLRP Family in Skeletal Tissues
Source: Biomolecules. 2020 Jan 3;10(1):80. doi: 10.3390/biom10010080 (PMC7023458; doi:10.3390/biom10010080)
Supplement: Supplementary file 1 [file biomolecules-10-00080-s001.pdf]

Supplementary materials

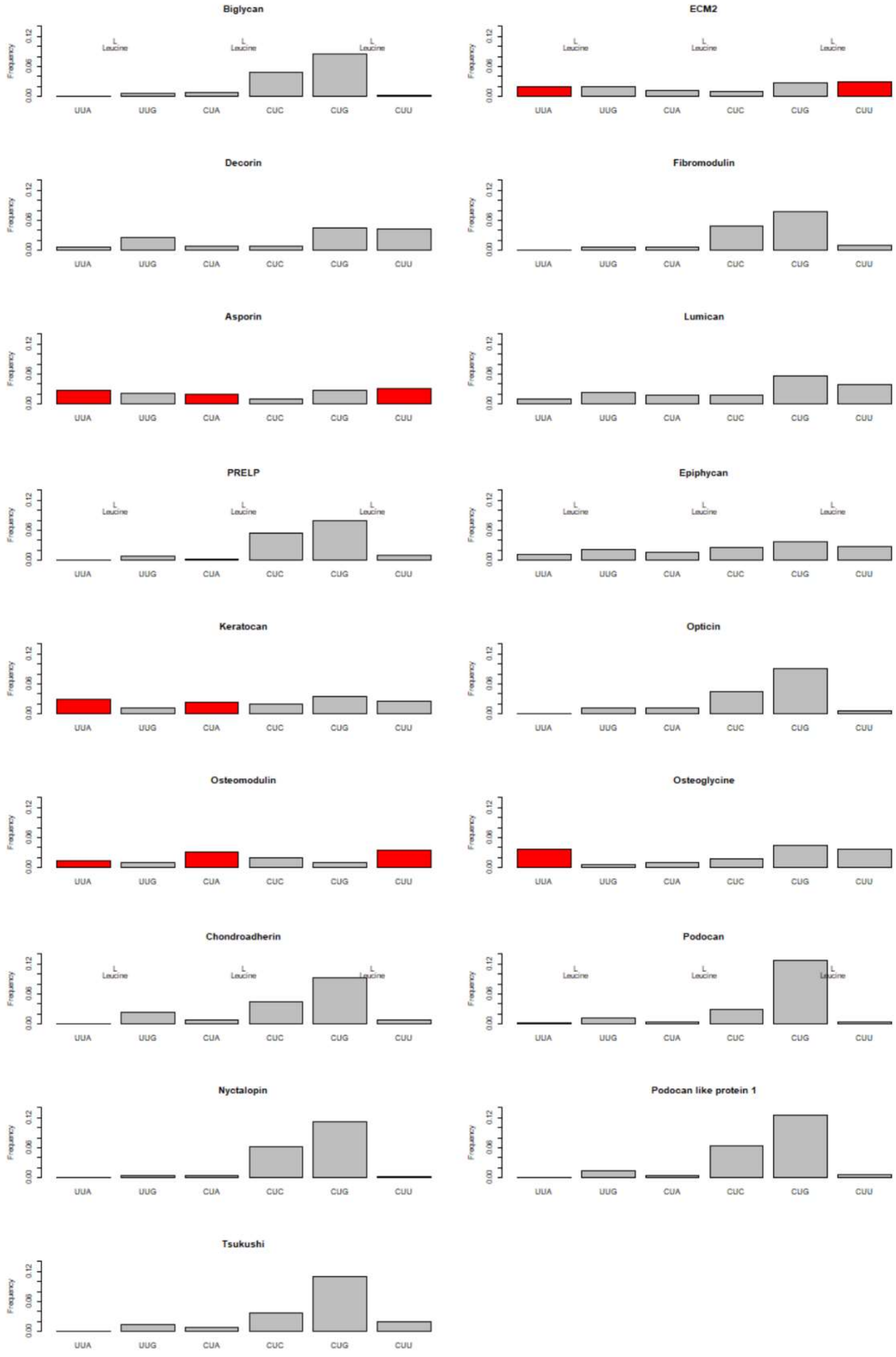

Figure S1. Comparison of codon usage frequencies for leucine (L) between protein members of the SLRP family. The red marked histograms show inverted codon usage bias as compared to most frequently encountered bias in humans, which are marked in gray.

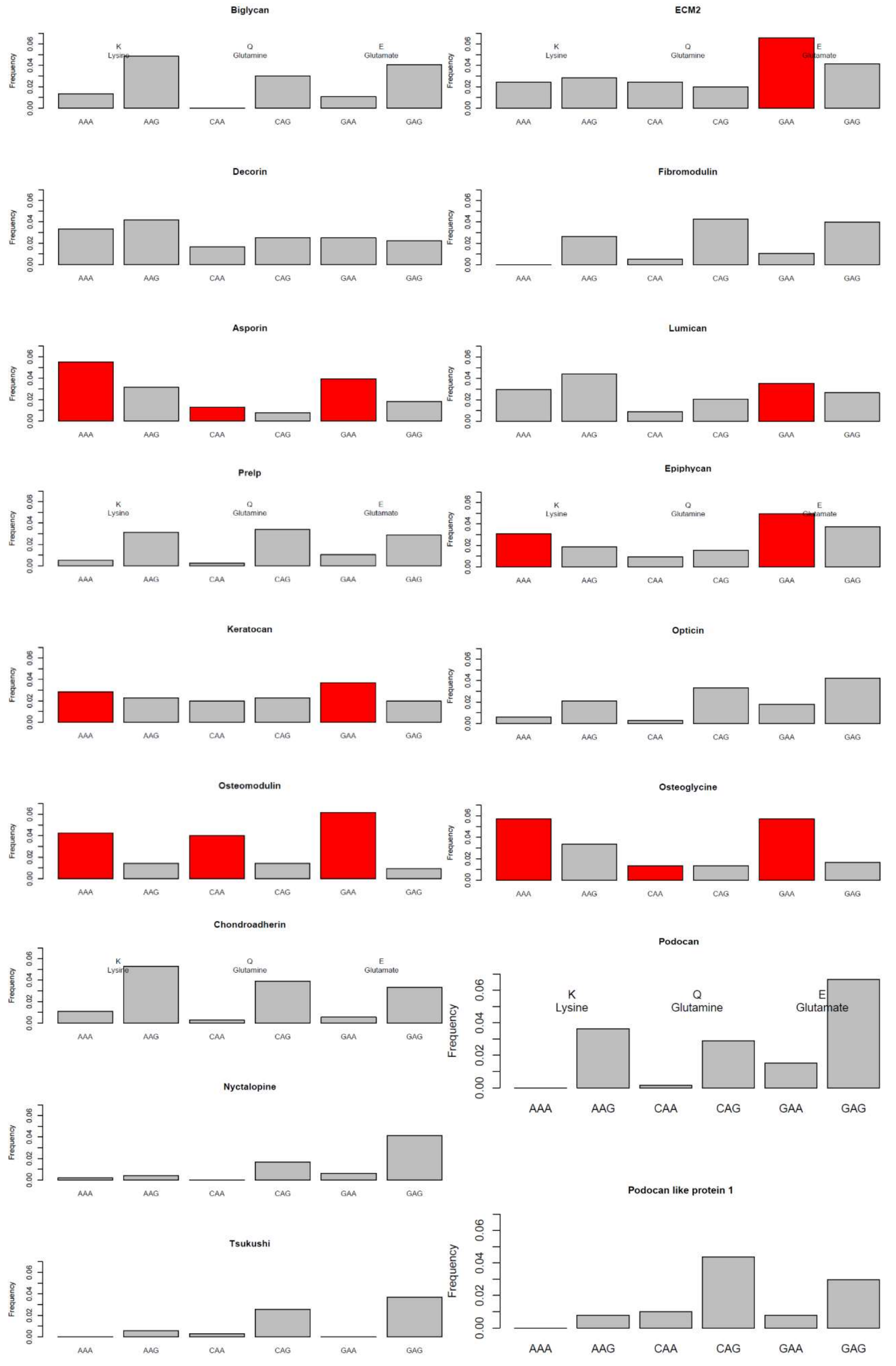

Figure S2. Comparison of codon usage frequencies for lysine (K), glutamine (Q), and glutamate (E) between protein members of the SLRP family. The red marked histograms show inverted codon usage bias as compared to most frequently encountered bias in humans, which are marked in gray.
